# The safety and effectiveness of clopidogrel versus aspirin in Kawasaki disease with mild-to-moderate liver injury

**DOI:** 10.1038/s41598-023-45647-6

**Published:** 2023-10-26

**Authors:** Lichao Gao, Wei Wang, Huafeng Wang, Zhufei Xu, Shulai Zhou, Zhimin Geng, Songling Fu, Chunhong Xie, Yiying Zhang, Yujia Wang, Fangqi Gong

**Affiliations:** 1 Department of Cardiology, Children’s Hospital, Zhejiang University School of Medicine, National Clinical Research Center for Child Health, No. 3333 Binsheng Road, Hangzhou, 310052 People’s Republic of China; 2 Department of Pulmonology, Children’s Hospital, Zhejiang University School of Medicine, National Clinical Research Center for Child Health, Hangzhou, China

**Keywords:** Cardiovascular diseases, Rheumatic diseases, Paediatric rheumatic diseases

## Abstract

Kawasaki disease can be combined with liver injury. As a mainstay treatment for Kawasaki disease, aspirin may cause liver injury. This study aimed to compare the safety and effectiveness of clopidogrel versus aspirin in Kawasaki disease with mild-to-moderate liver injury. This study retrospectively analysed 166 children with Kawasaki disease combined with mild-to-moderate liver injury. The children treated with clopidogrel were less likely to have aggravated liver injury than those treated with aspirin (n = 2/100 vs. n = 13/66, *P* < 0.001). The initial alanine aminotransferase value of the clopidogrel group was higher (131.5 [98.5, 167.5] vs. 96 [72, 133], *P* < 0.001), while the time of alanine aminotransferase recovery to normal was similar (5 [4, 7] vs. 4 [3, 7], *P* = 0.179). No significant fever differences observed between groups: 7.5 [6, 9] for aspirin vs. 7 [6, 8] for clopidogrel group, *P* = 0.064. The probability of nonresponse to intravenous immunoglobulin (n = 29/100 vs. n = 30/66, *P* = 0.030) and the days of hospitalization (n = 6 [4, 9] vs. n = 7 [5, 10], *P* = 0.007) in the clopidogrel group were less than those in the aspirin group. In conclusion, the application of clopidogrel is potentially superior to aspirin in Kawasaki disease combined with mild-to-moderate liver injury.

## Introduction

Kawasaki disease (KD), also known as mucocutaneous lymph node syndrome, is an acute systemic immune vasculitis^1^. It most commonly involves medium-sized arteries and can be combined with coronary artery disease, causing coronary artery dilatation or even large giant coronary aneurysms and thrombosis. At present, it is the major cause of acquired heart disease. Meanwhile, KD can also be combined with hepatobiliary injury and cause hepatitis, jaundice, cholecystitis and so on.

At present, the mainstay of initial treatment for KD is a single high dose of intravenous immunoglobulin (IVIG) together with aspirin. However, as a nonsteroidal anti-inflammatory drug, aspirin is thought to cause liver injury by itself. Therefore, for children with KD combined with severe liver function injury in the acute phase, aspirin is not recommended^2^. The utilization of aspirin in children with KD who exhibit mild-to-moderate liver function impairment continues to be a topic of debate. Meanwhile, an alternative antiplatelet choice for these patients is clopidogrel^2,3^. Is the use of clopidogrel compared to aspirin associated with less severe liver injury, a shorter recovery time from liver injury, a longer duration of fever, higher incidence of nonresponse to IVIG and more severe coronary artery lesion (CAL)? There are few studies on these aspects. To explore the application of antiplatelet agents for KD combined with mild-to-moderate liver injury, clinical data related to KD combined with liver injury were collected and analysed to provide a reference for treatment.

## Results

### Study population

**Figure 1:**
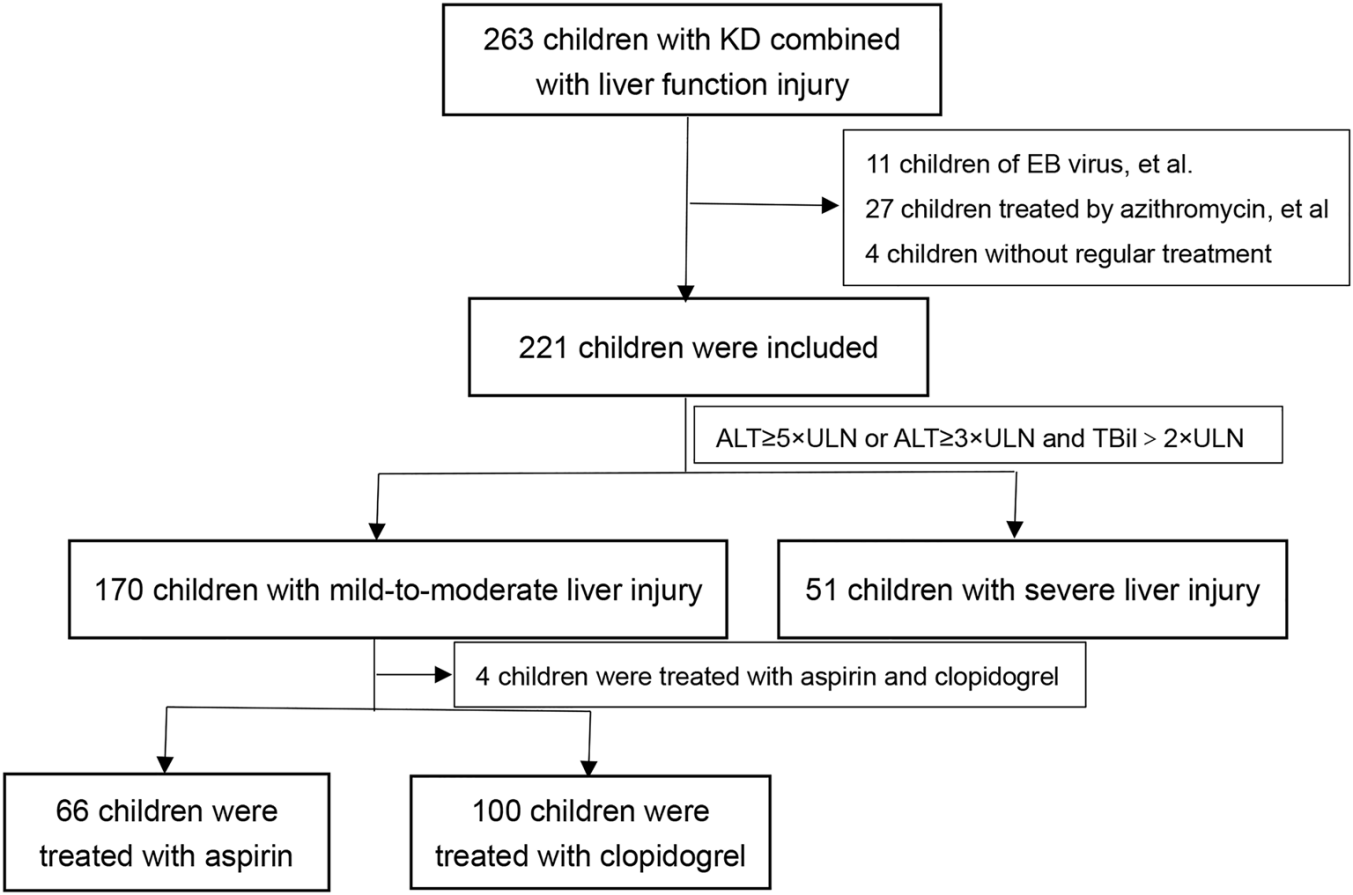
Flow of participants through the study.

Of a total of 263 children with KD combined with liver function injury from June 1, 2020, to December 31, 2021, 42 children were excluded, including 3 children with EB virus, 2 children with cytomegalovirus, 2 children with *Streptococcus pyogenes*, 1 child with hepatitis B virus, 1 child with respiratory syncytial virus, 1 child with adenovirus and 1 child with herpes simplex virus, 27 children treated with azithromycin and other drugs that might lead to liver injury, and 4 children without regular treatment outside the hospital, while 221 children were included. Among the 221 children, alanine aminotransferase (ALT) ≥ 5 × the upper limit of normal (ULN) was found in 49 children, and 2 children had ALT ≥ 3 × ULN and total bilirubin (TBIL) > 2 × ULN, for a total of 51 children in the severe liver injury group. In the remaining 170 children, 4 children were excluded because they were treated with aspirin and clopidogrel due to medium or large aneurysms discovered at the acute phase. Finally, 166 children with KD combined with mild-to-moderate liver injury were included in this study (Fig. 1).

### Patient characteristics and laboratory findings

The general information of the children is shown in Table 1. Of these 166 children, 96 (57.8%) were boys, with an average age of 28.5 [18.0, 47.3] months. All of them were Han Chinese. Fifty-nine (35.5%) children received second-line treatment with a second dose of IVIG, methylprednisolone or infliximab due to nonresponse to IVIG. Twenty-five (15.1%) children suffered from aneurysms, including 17 small aneurysms, 5 medium aneurysms and 3 large or giant aneurysms. The detailed characteristics of the participants who were diagnosed with complete and incomplete KD are shown in the supplemental data (Supplement Table 1).

**Table 1:** General information of the children.

Item	Number
Number of children	166
Number of infancy (% patients)	22 (13.3%)
Number of early childhood (% patients)	75 (45.2%)
Number of preschool age (% patients)	55 (33.1%)
Number of school-age or senior (% patients)	14 (8.4%)
Number of boys (% patients)	96 (57.8%)
Average age in month [min, max]	28.5 [5, 168]
Ethnicity	
Han Chinese (% patients)	166 (100%)
Average length of hospitalization in days [min, max]	6 [3, 34]
Number of nonresponse to IVIG (% patients)	59 (35.5%)
Number of Coronary artery lesions (% patients)	25 (15.1%)
Small aneurysms	17
Medium aneurysms	5
Large or giant aneurysms	3
Average value in ALT [max]	119 [247]
Average day for ALT recovery [min, max]	5 [2, 25]

*IVIG* intravenous immunoglobulin, *ALT* alanine aminotransferase, *infancy* < 1 year of age, *early childhood* 1–2 years of age, *preschool age* 3–5 years of age, *school-age or senior* 6–18 years of age, *small aneurysms* Z score ≥ 2.5 to < 5, *medium aneurysms* Z score ≥ 5 to < 10, with an absolute luminal dimension < 8 mm, *large and giant aneurysms* Z score ≥ 10 or absolute dimension ≥ 8 mm.

### Using aspirin or clopidogrel in patients with mild-to-moderate liver injury

**Figure 2:**
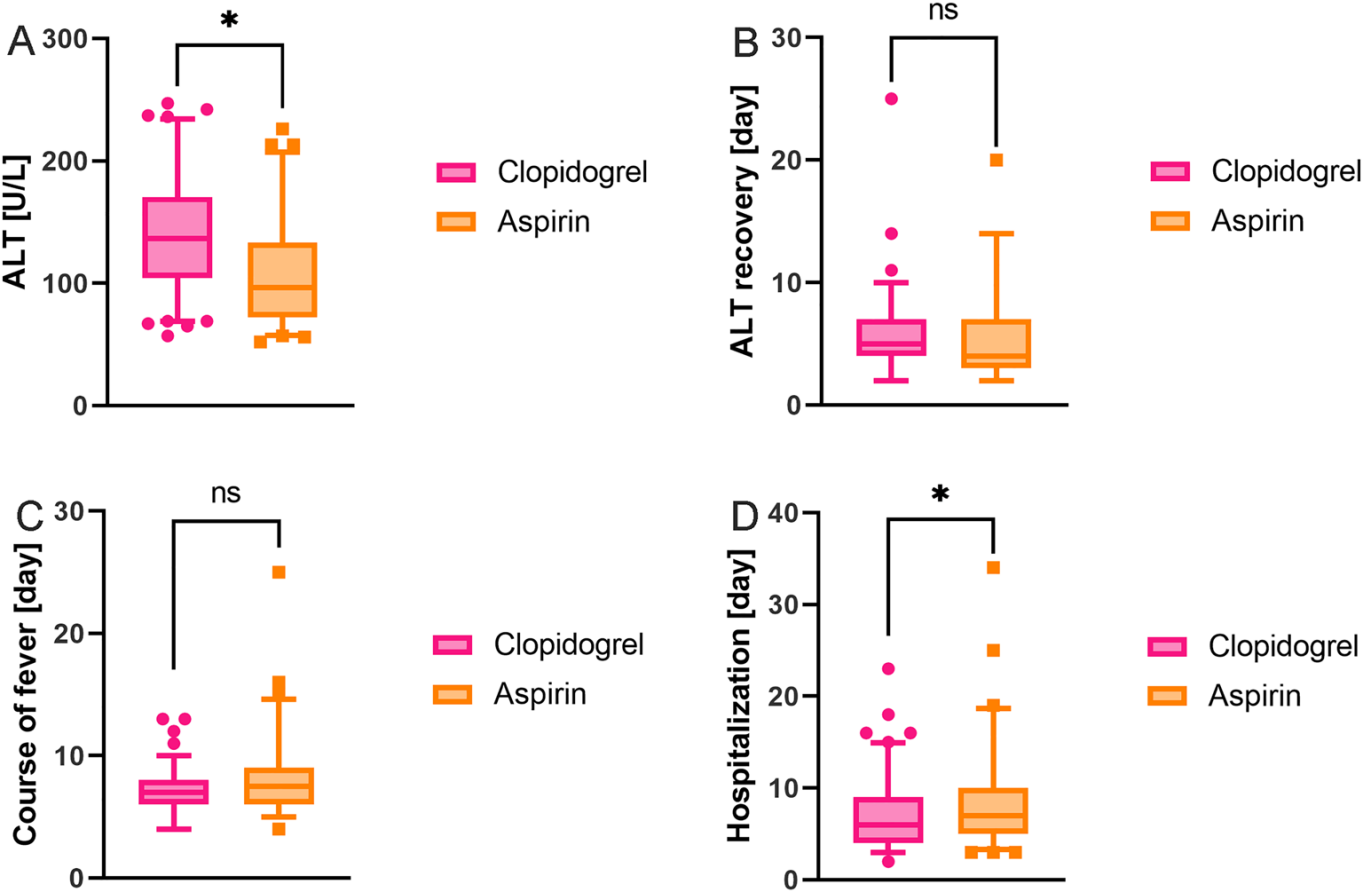
Comparison between aspirin and clopidogrel in patients with mild-to-moderate liver injury. (**A**) Comparison of the value of alanine aminotransferase (ALT) between the two groups; (**B**) Comparison of days of ALT recovery to normal between the two groups. (**C**) Comparison of the course of fever between the two groups; (**D**) Comparison of days of hospitalization between the two groups. *P < 0.05.

Among the remaining 166 children with mild-to-moderate liver injury, 66 children were treated with aspirin, and 100 children were treated with clopidogrel. There were no significant differences in age, sex, inflammatory indices, or cytokines between the two groups when comparing the children who used aspirin versus those who used clopidogrel (Table 2). We analysed the changes in ALT after treatment with IVIG and aspirin/clopidogrel in the two groups and found that the proportion of patients with elevated ALT levels was significantly higher in the aspirin group (n = 13/66) than in the clopidogrel group (n = 2/100) (Table 2). Although the initial ALT value of the clopidogrel group (131.5 [98.5, 167.5] vs. 96 [72, 133], *P* < 0.001) was higher (Fig. 2A), there was no significant difference in the time of ALT recovery to normal (5 [4, 7] vs. 4 [3, 7], *P* = 0.179) (Fig. 2B). The course of fever between the two groups was similar (7.5 [6, 9] in the aspirin group vs. 7 [6, 8] in the clopidogrel group, *P* = 0.064) (Fig. 2C), and the days of hospitalization in the clopidogrel group were less than those in the aspirin group (n = 6 [4, 9] vs. n = 7 [5, 10], *P* = 0.007) (Fig. 2D). Meanwhile, the probability of nonresponse to IVIG was lower in the clopidogrel group (n = 29/100 vs. n = 30/66), and the probability of CAL was similar between the two groups (n = 13/100 vs. n = 12/66) (Table 2).

**Table 2:** Comparison between aspirin and clopidogrel in patients with mild-to-moderate liver injury.

	Aspirin (n = 66)	Clopidogrel (n = 100)	*P* value
Age (month)	29 [16.75, 54.25]	28 [19.25, 44.75]	0.506
Number of boys (% patients)	41 (62.1%)	55 (55%)	0.363
ALT-elevated (% patients)*	13 (19.7%)	2 (2.0%)	< 0.001
Nonresponse to IVIG (% patients)*	30 (45.5%)	29 (29.0%)	0.030
CAL (% patients)	12 (18.2%)	13 (13%)	0.361
WBC (*10^9/L)	13.97 [10.17, 17.01]	14.54 [11.3, 17.84]	0.339
HB (g/L)	108 [99, 116]	110 [104, 117]	0.348
PLT (*10^9/L)	309.75 ± 82.28	313.16 ± 88.85	0.438
CRP (mg/L)	72.3 [39.63, 106.39]	76.94 [47.48, 106.55]	0.557
ESR (mm/h)	56.64 ± 21.93	60.36 ± 25.01	0.436
IL-2 (pg/ml)	3.1 [2.53, 3.93]	3.3 [2.7, 3.9]	0.279
IL-4 (pg/ml)	2.4 [1.7, 2.8]	2.5 [2, 2.9]	0.245
IL-6 (pg/ml)	131.8 [72.2, 337.83]	84.9 [39.3, 266.3]	0.077
IL-10 (pg/ml)	13.1 [6.08, 20.45]	9 [4.6, 26.6]	0.212
TNFα (pg/ml)	1.6 [1.13, 2.375]	1.5 [1, 2]	0.249
IFNγ (pg/ml)	4.55 [2.3, 8.93]	3.6 [2, 6.4]	0.259

*ALT* alanine aminotransferase, *IVIG* intravenous immunoglobulin, *CAL* coronary artery lesions, *WBC* white blood cell, *HB* haemoglobin, *PLT* platelets, *CRP* c-reactive protein, ESR erythrocyte sedimentation rate, *IL* interleukin, *TNF* tumour necrosis factor, *IFN* interferon.

*P < 0.05.

#### Factors associated with the course of fever

In multivariate linear regression analysis, day of illness at initial treatment and platelet count were the factor independently associated with the course of fever after controlling for other variables (Table 3). The application of aspirin or clopidogrel as antiplatelet agents was not associated with the course of fever (*P* = 0.114).

**Table 3:** Multivariate Linear Regression analyses for the course of fever.

Effect	Estimate	SE	95% CI		*P*
			LL	UL	
Intercept	6.665	6.252	− 5.683	19.013	0.288
Drugs^a^	− 0.522	0.329	− 1.171	0.127	0.114
Day of illness at initial treatment	1.036	0.122	0.796	1.276	< 0.001
Serum sodium	− 0.023	0.042	− 0.106	0.061	0.593
Serum aspartate aminotransferase	0.002	0.002	− 0.002	0.007	0.324
Percentage of WBC representing neutrophils^b^	0.025	0.014	− 0.003	0.053	0.083
C-reactive protein	− 0.002	0.004	− 0.009	0.006	0.651
Platelet count	− 0.006	0.002	− 0.010	− 0.002	0.001
Age in months	0.001	0.007	− 0.012	0.014	0.837

Model: F = 15.115, *P* < 0.001; R^2^ = 0.435.

*CI* confidence interval, *LL* lower limit, *UL* upper limit.

^a^1 = Aspirin; 2 = Clopidogrel.

^b^*WBC* white blood cells.

## Discussion

Kawasaki disease is a systemic inflammatory disease involving all medium-sized arteries and multiple organs. In addition to coronary aneurysms, hepatic dysfunction is also a common complication at the acute phase of KD^4^. Aspirin has been one of the standard therapies for KD patients, but it has some adverse effects, such as hepatic toxicity and potential risk for inducing Reye syndrome. In this retrospective study, we investigated the safety and efficacy of clopidogrel versus aspirin in KD with mild-to-moderate liver injury. In this study, we found that children treated with aspirin were more likely to have aggravated liver injury than those treated with clopidogrel. The initial ALT value of the clopidogrel group was higher, while the time of ALT recovery to normal was similar. The course of fever between the two groups was similar. Meanwhile, the probability of nonresponse to IVIG and the days of hospitalization in the clopidogrel group were less than those in the aspirin group. This suggests that the application of clopidogrel is potentially superior to aspirin in KD combined with mild-to-moderate liver injury.

Mild to moderate serum aminotransferase or gamma-glutamyltranspeptidase elevation was found in 40% to 60% of the children, and 10% of the children showed mild hyperbilirubinemia^5,6^. Hepatic pathology found inflammatory cell infiltration, Kupffer cell proliferation and/or swelling, steatosis and severe congestion in the blood sinus and header area^6^. The mechanism of abnormal hepatic function in KD has not been determined and may be related to systemic inflammation, small- and medium-vessel vasculitis, congestive heart failure secondary to myocarditis, nonsteroidal anti-inflammatory and antipyretic, toxin-mediated effects, or the combination of those events. Relevant studies have shown that children with KD combined with hepatic dysfunction show an increasing probability of nonresponse to IVIG and CAL^4,7–9^. In the study, the proportion of nonresponse to IVIG and CAL in hepatic dysfunction was 37.1% and 17.6%, respectively, which were significantly higher than the average value of children with KD that has been previously reported^3,10,11^. This was also higher than the result reported by Eladawy M et al.^6^, in which the rate of nonresponse to IVIG was 22%, and the rate of CAL was 10%. This may be because many patients with mild disease went home after outpatient treatment with IVIG during this period, resulting in a high proportion of inpatients who did not respond to IVIG and CAL. Liver function lesions commonly occur during the acute phase of KD, and most children can recover after approximately 6 days of initial treatment.

The determination of ALT and aspartate aminotransferase (AST) values was classically used to evaluate liver function. AST was useful initially but was found to be nonspecific for heart muscle. The serum level of ALT has been considered the leading marker of liver injury^12^, including multiple aetiologies from viral hepatitis to fatty liver disease. Here, we chose the criterion of ALT greater than 50 U/L as one of the inclusion criteria. However, elevated ALT levels are not a metrical criterion for clinical severity. In view of the high sensitivity of ALT and the high specificity of TBIL, the combined application of ALT and TBIL is helpful to better judge liver injury^13^. There is no recognized uniform identification of severe liver injury. The severity of the National Cancer Institute general toxicity standard from 1982 to 2012 showed that^13^ mild (ALT > 1–2.5 × ULN), moderate (2.5–5 × ULN), severe (ALT > 5–20 × ULN), and life-threatening (ALT > 20 × ULN). The Asia Pacific Association of Study of Liver consensus guidelines^14^ in 2021 defined that at least one of the following conditions in drug-induced liver injury should be met: (1) ALT ≥ 5 × ULN; (2) alkaline phosphatase (ALP) ≥ 2 × ULN; and (3) ALT ≥ 3 × ULN and total bilirubin > 2 × ULN. ALP in children depends on age, as it might be affected by bone growth, and the value will be higher than that in adults^15^. As a result, one of the following conditions was defined as severe liver injury for this study: ALT ≥ 5 × ULN or ALT ≥ 3 × ULN and total bilirubin (TBIL) > 2 × ULN. It was found that various liver function indices were affected in the severe liver injury group, and the days of hospitalization and ALT recovery to normal were also longer (Supplement Table 2), which suggested that the definition of severe liver injury according to the above method shows a certain justification.

Antiplatelet drugs are important components of the treatment of KD. Commonly used antiplatelet drugs included aspirin, clopidogrel, flurbiprofen, dipyridamole, and ticlopidine. According to the Japanese Circulation Society/Japanese Society for Cardiovascular Surgery 2020 Guideline on Diagnosis and Management of Cardiovascular Sequelae in KD^3^, there is no indication to use flurbiprofen for KD, and dipyridamole was mainly used for angina pectoris or myocardial infarction. Cyclooxygenase-1 (COX-1) could be inhibited by aspirin through acetylation, and the production of thromboxane A2, which promotes platelet aggregation, could also be inhibited, which showed an antiplatelet effect^16^. Moreover, previous allergy history, peptic ulcer, bleeding tendency, and asthma were contraindications according to the packaging instructions. Careful administration was required for severe liver injury, but it was not an absolute contraindication. The adenosine diphosphate receptor (P2Y12) coupled by inhibitory G-protein could be inhibited by clopidogrel, and then the antiplatelet effect could be induced by promoting the increase in cAMP concentration by inhibiting adenylate cyclase^17^. The usage of clopidogrel in children was 0.2–1.0 mg/kg/day, once per day. However, it is also not recommended for children with severe liver injury. In the HOST-EXAM trial, it was found that clopidogrel was superior to aspirin in preventing the primary composite thrombotic endpoint and the bleeding endpoint after percutaneous coronary intervention^18^.

In our centre, aspirin and clopidogrel were used for antiplatelet therapy in KD with mild-to-moderate liver injury. Moderate doses (30-50 mg/kg) of aspirin are believed to have anti-inflammatory and antipyretic effects. In order to compare whether the use of clopidogrel can prolong the duration of fever in children with KD, we compared the course of fever in the two groups and found that the course of fever in the clopidogrel group did not increase. To ensure the credibility of the results, we further conducted a multivariate analysis. Few studies investigated the risk factors for prolonged fever of KD, but some risk factors for IVIG unresponsiveness have been established from previous clinical studies. We thought using those risk factors as the predictors of prolonged febrile days would be acceptable. Here, We had used the predictor of the drug (aspirin or clopidogrel) as a dummy number, and screened the risk factors for IVIG unresponsiveness based on the Kobayashi score^19^: day of illness at initial treatment, serum sodium, serum AST, percentage of white blood cells representing neutrophils, C-reactive protein, platelet count and age in months, using multiple regression analysis to analyze the course of fever. After controlling potential risk factors, there was no significant difference in the course of fever between the two groups. This result indicated that clopidogrel therapy does not increase the duration of fever in patients with KD with mild-to-moderate liver injury.

When comparing aspirin with clopidogrel in the treatment of children with mild-to-moderate liver injury, it was shown that there were no significant differences in age, sex, inflammatory index, course of fever or proportion of CAL between the two groups, which indicated that the degree of vasculitis was similar. In our previous study^20^, we found that the levels of IL-6, IL-10, TNFα, and IFNγ were helpful for predicting KD prognosis. In this study, there was no significant difference in the levels of IL-6, IL-10, TNFα, and IFNγ between the clopidogrel and aspirin groups, which might once again confirm the similar degree of vasculitis in the two groups. Although the initial ALT value of the clopidogrel group was higher, there was no significant difference in the time of ALT recovery to normal, which suggests that the recovery of the clopidogrel group was relatively quick. The proportion of ALT elevation was significantly higher in children who received IVIG combined with aspirin than in those who received IVIG combined with clopidogrel, indicating that aspirin was more likely to cause liver damage. It was found that the rate of nonresponse to IVIG in the children treated with clopidogrel was lower than that in children treated with aspirin. We reviewed the literature and found that the following theories might explain this result. The P2Y12 signalling pathway serves as an important autocrine and paracrine feedback loop with a central role in amplifying platelet activation^21^. Activated platelets interact with leukocytes, triggering intercellular signalling events that lead to thrombus formation and the massive synthesis of inflammatory mediators^22^. Clopidogrel could enhance the bioavailability of endothelial nitric oxide and inhibit platelet activation, platelet degranulation, platelet-leukocyte aggregate formation, inflammatory cytokine expression, and tissue factor expression^23^. Compared with aspirin, clopidogrel was more effective in reducing the formation of platelet-leukocyte aggregates^24^. In conclusion, it was believed that the application of clopidogrel might be better than aspirin in KD with mild-to-moderate liver injury.

This retrospective study was limited by its single-centre design and relatively small sample size, which may limit the generalizability of the results to other populations. The decision on which antiplatelet drug to use depended partly on the doctor’s discretion, which could lead to a possible selection bias. As the total number of patients who met the criteria for sustained elevated ALT levels was only 15, which did not meet the minimum number for multivariable logistic regression calculations based on the "rule of thumb" (10 events per predictor), we were unable to conduct a multivariable analysis for this outcome. To confirm these results, we may need to further expand the sample size to clarify this result. Moreover, a multicentre randomized controlled clinical study may be required if necessary.

In summary, liver injury commonly occurred in the acute phase of KD, and most children recovered to normal after initial treatment with IVIG. In children with KD combined with mild-to-moderate liver injury, the use of clopidogrel may offer potential advantages over aspirin. Our study suggests that clopidogrel may be a viable antiplatelet therapy option for these patients, but further research is needed to confirm its preferred status.

## Methods

### Inclusion criteria

Children diagnosed with KD combined with liver function injury (ALT greater than 50 U/L) at the acute phase and who were hospitalized in the Children's Hospital of Zhejiang University School of Medicine from June 1, 2020, to December 31, 2021, were enrolled retrospectively, and the children were subjected to control analysis in combination with medical records, laboratory results and echocardiography tests.

The diagnostic criteria of KD included: complete KD: at least 5 of the following 6 main clinical characteristics: fever > 5 days; bilateral bulbar conjunctival injection; changes in the lips and oral cavity: reddening of the lips, strawberry tongue, diffuse injection of oral and pharyngeal mucosae; rash (including redness at the site of BCG inoculation); changes in the peripheral extremities: (initial stage) reddening of the palms and soles, oedema; (convalescent stage) periungual desquamation; and nonsuppurative cervical lymphadenopathy. For incomplete KD, children with fever ≥ 5 days but with less than 4 of the the main clinical characteristics were evaluated as incomplete KD according to the process in the 2017 American Heart Association scientific statement^25^. Nonresponse to IVIG was defined as a body temperature higher than 38 °C at least 36 h after the first IVIG infusion or a fever again within 2 weeks (mostly 2–7 days) after drug use, and there was at least one main clinical manifestation of KD after other possible causes of fever were excluded. CAL was based on the Z score classification, which was diagnosed using an echocardiogram, and a Z score greater than 2.5 was considered abnormal: no involvement (Z score always < 2), dilation only (Z score ≥ 2 but < 2.5, or a decrease in Z score during follow-up ≥ 1), small aneurysms (Z score ≥ 2.5 to < 5), medium aneurysms (Z score ≥ 5 to < 10, with an absolute luminal dimension < 8 mm), and large and giant aneurysms (Z score ≥ 10 or absolute dimension ≥ 8 mm)^3,11,25^.

The diagnostic criteria of liver function injury were as follows: ALT greater than 50 U/L. As there was no definition of severe liver injury in KD, we distinguished whether there was severe liver injury according to the consensus guidelines of the Asia Pacific Society of Hepatology and National Cancer Institute common toxicity criteria grades of severity^13,14^. One of the following conditions was defined for severe liver injury: ALT ≥ 5 × ULN or ALT ≥ 3 × ULN and TBIL > 2 × ULN.

Initial treatment of KD: Once KD has been diagnosed, a single high dose of IVIG (2 g/kg) and aspirin (initially 30–50 mg/d orally in 3 divided doses; after 48–72 h of fever reduction, reduce the dose to 3–5 mg/kg by oral administration) should be used except in the following situations: 1. concurrent infection with influenza or varicella virus; 2. in combination with severe liver injury. 3. Aspirin allergy. When aspirin was not suitable in the above three conditions, clopidogrel was used. In our centre, both aspirin and clopidogrel were selected for the treatment of patients with KD with mild-to-moderate liver injury.

### Exclusion criteria

Outpatient cases were not included. Children without regular treatment outside the hospital (for example, methylprednisolone was used before IVIG) combined with diseases that may cause liver injury and drugs that might lead to liver injury were excluded.

### Methodology

The clinical characteristics (age, sex, liver function indices, inflammatory indices, cytokines, clinical manifestations, complications and echocardiographic data) were collected based on medical records. Liver function indices were collected at the time of admission, after 5–7 days of initial treatment and when the ALT value reached its highest level during the course of the disease. Inflammation indices and cytokines were collected at admission, and all echocardiographic data were analysed in detail for assessment of CAL.

According to the criteria of severe liver injury, the patients were divided into a mild-to-moderate liver injury group (n = 170) and a severe liver injury group (n = 51). In the mild-to-moderate liver injury group, the patients were divided into an aspirin group (n = 66) and a clopidogrel group (n = 100) by the antiplatelet agents used.

The primary outcome was the proportion of patients with elevated ALT levels after initial treatment and the course of fever. Secondary outcomes included the time of ALT recovery, the days of hospitalization, the probability of nonresponse to IVIG, and the probability of CAL.

### Statistical methods

The quantitative data were evaluated using the *one-sample Kolmogorov–Smirnov test* to determine whether they followed a normal distribution. The measurement data conforming to a normal distribution are presented as the *mean* ± *standard deviation (SD)*, and independent sample t tests were used for hypothesis testing. The measurement data that did not follow a normal distribution are represented as the *median [interquartile range]*, and nonparametric tests (*Mann–Whitney U* test, *Kruskal–Wallis H* test) were used for hypothesis testing. Counting data were represented as the number of children and percentage (%), and chi-square tests were used for hypothesis testing. Multivariate analysis was conducted using multiple regression analysis. Statistical analysis was performed using SPSS 16.0 software. A two-sided, and *P* value < 0.05 was considered statistically significant.

### Ethics declarations

This retrospective study involving human participants was in accordance with the ethical standards of the institutional and national research committee and with the 1964 Helsinki Declaration and its later amendments or comparable ethical standards. The medical ethics committee of the Children's Hospital of Zhejiang University School of Medicine approved this study (NO.: 2022-IRB-118). Since the information was anonymized and the submission did not include images that may identify the person, the medical ethics committee of the Children's Hospital of Zhejiang University School of Medicine waived the need for individual consent forms.

### Supplementary Information

Supplementary Table 1.

Supplementary Table 2.

## Data Availability

Anonymized research data are available from the corresponding author upon reasonable request.
