# Supplementary material for: The safety and effectiveness of clopidogrel versus aspirin in Kawasaki disease with mild-to-moderate liver injury
Source: Sci Rep. 2023 Oct 26;13:18324. doi: 10.1038/s41598-023-45647-6 (PMC10603134; doi:10.1038/s41598-023-45647-6)
Supplement: Supplementary file 2 — Supplementary Table 2. [file 41598_2023_45647_MOESM2_ESM.pdf]

The safety and effectiveness of clopidogrel versus aspirin in Kawasaki disease with mild-to-moderate liver injury

Lichao Gao*, Wei Wang*, Huafeng Wang, Zhufei Xu, Shulai Zhou, Zhimin Geng, Songling Fu, Chunhong Xie, Yiying Zhang, Yujia Wang#, Fangqi Gong#

Supplement Table 2. Comparison between the severe liver injury group and the mild-to-moderate liver injury group

	mild-to-moderate liver injury (n=170)	severe liver injury (n=51)	*P* value
Age (month)	28 [18, 46.25]	33 [16, 45]	0.735
Number of boys (% patients)	97 (57.1%)	36 (70.6%)	0.083
Hospitalization (day)*	6.5 [5, 9.25]	8 [6, 12]	0.028
Nonresponse to IVIG (% patients)	61 (35.9%)	21 (41.2%)	0.492
CAL (% patients)	29 (17.1%)	10 (19.6%)	0.675
ALT	118.50 [88.75, 160.25]	373.00 [287.00,487.00]	<0.001
ALT recovery (day)*	5 [4, 7]	8 [7, 10]	<0.001
AST (U/L)*	48 [30.75, 80.5]	114 [47, 184]	<0.001
DBILI (umol/L)*	2.4 [1.48, 4.23]	4.6 [2.1, 11.8]	<0.001
IBILI (umol/L)*	4.8 [2.88, 8.08]	8.2 [4.8, 15.5]	<0.001
Albumin (g/L)*	32.59±4.71	35.18±3.36	<0.001
γ-GT (U/L)*	92 [52.75, 139.5]	153 [114, 239]	<0.001
ALP (U/L)*	213.5 [173.5, 270]	325 [206, 387]	<0.001
TBA (umol/L)*	9.55 [5.08, 22.93]	38.7 [7.8, 159.3]	<0.001

Prealbumin (mg/L)*	63.45 [47.7, 79.73]	79.6 [64.8, 97.5]	<0.001

*$P<0.05$, IVIG: intravenous immunoglobulin, CAL: coronary artery lesion, ALT: alanine aminotransferase, AST: aspartate aminotransferase, DBILI: direct bilirubin, IBILI: indirect bilirubin, γ-GT: gamma-glutamyltransferase, ALP: alkaline phosphatase, TBA: total bile acid.
